# Report of the Education-Committee of the General Medical Council

**Published:** 1890-12

**Authors:** 


### Reviews of Books.

---

*Report of the Education-Committee of the General Medical Council.* Printed by SPOTTISWOODE & CO. 1890.

"A grand opportunity missed," is the depressing verdict which a careful perusal of the above begets.

After 1892 the duration of medical study is to be five years. If these five years had been devoted to genuine medical study, we should have had nothing but congratulations to offer; but as the first of these five years is devoted to studies which are not medical, and as the last year is to be given over to pursuits for which English medical institutions are not prepared, we are driven to adverse criticism. There are, in fact, but three solid years of medical study provided for in the new scheme. And these three years are to be softened down; the student is to be lectured at less. But, as a counterpoise to this freedom, the Committee recommend an increased attention to spelling (!) among those who desire to become a "member of a profession whose position is so high."

The Report of the Committee is so far from coming up to the standard we had expected, that we took the trouble to look up the personal record of the gentlemen who drew it up. The chairman, Dr. Struthers, late Professor of Anatomy at Aberdeen, is a tried and valiant fighter in the cause of medical education from the scientific side, of which alone he has experience. Unfortunately he is a profound admirer of the German and French systems, and still more unfortunately he was chairman. From Dublin we have Sir John Banks and Dr. Kidd, who have each been nearly half a century in the profession, and whose opinions should have at least historical value; and the Rev. Samuel Haughton, who possesses every conceivable capacity except the not unimportant one of teaching or practising medicine. Then we have Dr. Glover, of London, a gentleman who has not made his influence felt either in medical practice or in medical education; Dr. Batty Tuke, an authority on Lunacy, from

Edinburgh; Mr. Wheelhouse, of Leeds, an able practitioner of forty years' standing; and last, but not least, Mr. Mitchell Banks, of Liverpool, and Dr. Leishman, of Glasgow.

Now, these men, all of them, may be leading authorities on medical education, but, with one or two exceptions, they have never shown it. They are mostly men of standing in the profession, by duration as well as by repute. They have not all the same bent: they do not clash. Dr. Leishman might get a special winter session for Midwifery, if Dr. Haughton got one on Physics. Mr. Wheelhouse might secure a return to his favoured system of pupilage, as a counterpoise to Dr. Batty Tuke's stipulation for attendance on properly registered dispensaries and "Institutions." There is not an Arts graduate on the Committee (according to the *Medical Directory*) except Dr. Haughton, and with him, unfortunately, general culture is not united to medical practice.

These are the men. We have three or four aged physicians, a lunacy doctor, a couple of surgeons, an obstetrician, and a chairman who, being chairman, does not count. They meet and pass a comprehensive scheme of medical education; but they meet in nothing else. They pass an average which is an average of extremes, and therefore valueless. Each individual carries his own point; and the chairman, who has scarcely practised his profession at all, sees them all satisfied, and is satisfied also.

For a detailed criticism of the scheme we have no space. Nor is such detailed criticism necessary, for a few of its provisions are enough to condemn the whole.

And first as to the preliminary examination. It comes into operation on January 1st, 1892; it might have been in operation for a couple of centuries. "English Language, including Grammar and Composition"! The President of the Council in 1882 groans over the "persistent frequency of examples of bad spelling." Well, what does he expect? Latin of the most elementary; Mathematics comprising Algebra as far as Simple Equations; the first three books of Euclid, and a few optional subjects of no value. Here is the culture-test necessary for the aspirant who would enter what is grandiosely described as one of "The Learned Professions"; and here our "Education-Committee" lets it remain. It is a crying evil that at the threshold of the medical profession the entrance is made ridiculously easy, easier certainly than to the counting-house of a linen-draper. The Committee must have known this—the remarks of the President show that they ought to have known it—and they

20

seek to make no improvement. We cannot turn half-educated boys into cultured medical men. Unless the Registration Examination is made to be an index of general culture, the President must continue to lament ignorance of ordinary spelling and composition on the part of medical students; and teachers must continue to fail to raise the standard of medical knowledge. It is unjust to the students also: they are let in too easily, and they get out of their studies with too much difficulty; there are too frequent rejections at the medical examinations, and too many absolute failures. All this might have been amended if the Education-Committee had distinctly raised the standard of the Registration Examinations.

The first year of actual study is spent in studying the elements of science, which the student should have acquired before registration. To import general science into the purely medical curriculum is both unnecessary and deleterious. It is unnecessary, because nowadays in all teaching medical centres there are abundant outside opportunities for acquiring a knowledge of the scientific subjects named; and it is deleterious, because it robs the student of a year's work which, while being called "medical," is not medical at all. Four years' medical study should not be described as five. These subjects should be examined for in the Entrance or Registration Examination, but they should not be included among medical studies.

The next three years are to be devoted to the serious study of medicine; these we pass over. Let us see what is to be made of the fifth. This "should be devoted to clinical work at one or more of such Public Hospitals or Dispensaries, British or foreign, as may be recognised by any of the Medical Authorities mentioned in Schedule A of the Medical Act (1858); provided that of this year six months may be passed as a pupil to a Registered Practitioner holding a Public appointment or possessing such opportunities of imparting practical knowledge as shall be satisfactory to the Medical Authorities." Theoretically, these regulations for the fifth year seem to be sensible; but practically, we fear they will not work. The student is to devote his whole day to clinical work for six months at a hospital or allied institution, and, permissibly, for six months with a registered practitioner. To safeguard against the palpable dangers of pupilage to the registered practitioner will, at the outset, be very difficult. How would such a pupil always differ from the objectionable "unqualified assistant"? And is it not conceivable that such pupilage will simply be signified by the suggestive term

"signing up"? In the few cases where busy men would care to have private pupils and deal honestly and capably by them, is it expected that they would do so free of charge; or, rather, would such apprenticeship not be smartly charged for? If there is a demand there may be a supply, but the exchange value will be high.

As to the hospital clinical work, we fear that this is impossible, unless the rules of our institutions in Great Britain are greatly modified. Here our hospitals have many officers, with few beds; the hospital work is done during one or two hours of the day; during the rest of the day the senior officers are supposed to be earning their daily bread. The wards are then left in the charge of young residents. The clinical instruction, to occupy the whole day, must either be left to these assistants, or we must have a new set of officers, who can have a greater command of beds and more time to devote to them. In other words, they must be paid. On the Continent the system suggested is in force, but the whole hospital system is in keeping. In this country the hospital system must either be adapted to the continental, or the scheme suggested must be a failure. Unless Government takes over our hospitals and pays their medical men, giving them services of beds which occupy half their working time, we fear that to occupy the whole day of the student in clinical hospital work is impossible. In our largest schools, with comparatively few patients, the scheme is manifestly impossible; it would be very difficult to carry it out even where the schools are small and the clinical material abundant. Whatever may happen in the future, our means of working this part of the scheme at present are certainly inadequate.

There are several other details in the scheme open to adverse criticism, but we have said enough to show our general feeling about it. We believe it to be, not only no improvement, but a distinct retrogression. The time was ripe for a change, and a change that was to be an improvement would have been gladly welcomed on all hands. Education tests are advancing on all sides; the education test for medicine, universally admitted to be too low, is left stationary. Medical knowledge increases in bulk and complexity, and demands more general culture and better brains to master it. We import into the years available for medical education tuition in elementary sciences which should be the property of every schoolboy desiring to enter medicine. And, finally, a scheme of special clinical instruction is devised which, on the one hand, must upset and displace the system of prolonged clinical study at present in existence, and, on the other, cannot be carried out under existing plans of hospital management.

What better can be said of such a scheme than that it is bad? It is true that we have ourselves to blame. We have put men who are honour-worthy into places of honour; we are clearly wrong in expecting that these men, already past their meridian, shall also lead us in new paths towards improvement. They lose their way and hark back on old paths, familiar to them, but now grown over with weeds and abandoned. The pity is that they can compel the young and the vigorous, who have discovered better ways, to follow in their courses.

